# Overexpression of Lifeact in the *C. elegans* body wall muscle causes sarcomere disorganization and embryonic or larval lethality

**DOI:** 10.3389/fcell.2024.1504980

**Published:** 2024-11-13

**Authors:** Shoichiro Ono

**Affiliations:** ^1^ Departments of Pathology and Cell Biology, Emory University School of Medicine, Atlanta, GA, United States; ^2^ Winship Cancer Institute, Emory University School of Medicine, Atlanta, GA, United States

**Keywords:** actin, muscle, sarcomere, thin filaments, *Caenorhabditis elegans*

## Abstract

Lifeact is a short peptide that is widely utilized as a probe for actin filaments in live imaging. However, high concentrations of Lifeact can alter actin filament dynamics and cause artificial modifications to the actin cytoskeleton. Here, I evaluated *Caenorhabditis elegans* strains expressing Lifeact fused to fluorescent proteins in the body wall muscle. I found that, while low-level expression of Lifeact from a single-copy transgene was appropriate for labeling sarcomeric actin filaments, overexpression of Lifeact from an extrachromosomal array causes severe disorganization of muscle sarcomeres and lethality at an embryonic or larval stage. Therefore, for imaging studies in *C. elegans*, Lifeact needs to be kept at a low level by proper management of the expression system.

## Introduction

Actin is one of the major cytoskeletal proteins and it plays important roles in many cell biological events (Pollard, 2016). The actin cytoskeleton is constantly reorganized, and its dynamic behaviors are often critical for its functions. The development of genetically encodable actin probes in combination with fluorescent proteins as fusion partners has dramatically advanced the field of analyzing the dynamics of actin filaments in living cells (Melak et al., 2017). Lifeact, a 17-amino-acid peptide from yeast Abp140, is one of the most utilized F-actin probes for live imaging (Riedl et al., 2008). Lifeact labels actin filaments in cells from many different species but has some limitations, such as not detecting stress-induced actin-cofilin rods (Munsie et al., 2009) and poorly labeling certain filopodia and lamellar actin networks (Belin et al., 2014).

However, the major disadvantage is the detrimental effects of overexpressed Lifeact on the actin cytoskeleton. Overexpression of Lifeact causes F-actin disorganization and sterility in the *Drosophila* ovary (Spracklen et al., 2014), cardiac dysfunction in the zebrafish heart (Xu and Du, 2021), F-actin stabilization in the *Arabidopsis* root (van der Honing et al., 2011), prolonged endocytosis and cytokinesis in fission yeast (Courtemanche et al., 2016), and alterations in nuclear and cytoplasmic actin filaments in cultured mammalian cells (Du et al., 2015; Flores et al., 2019). In *in vitro* experiments, Lifeact alters rates of nucleation and elongation of actin filaments and inhibits filament severing by cofilin (Courtemanche et al., 2016), indicating that excessive Lifeact can artificially modify actin filament dynamics. Nonetheless, when Lifeact expression is managed at low levels, it is still a very useful F-actin probe for live imaging (Courtemanche et al., 2016; Alvarez et al., 2024; Wirshing and Goode, 2024).

In this study, I evaluated Lifeact as a probe for actin filaments in the body wall muscle of the nematode *Caenorhabditis elegans*, in which actin and myosin are organized in a sarcomeric pattern (Benian and Epstein, 2011; Ono, 2014). Since many structural and regulatory proteins for assembly and maintenance of sarcomeric actin filaments are conserved among vertebrates and invertebrates (Ono, 2010), the *C. elegans* body wall muscle is an excellent model for studies on the structure and function of the striated muscle. One of the major advantages of *C. elegans* is its transparent body, allowing live fluorescence imaging of the body wall muscle in intact animals (Ghosh and Hope, 2010). Previously, we have reported that green fluorescent protein (GFP)-tagged actin (GFP::ACT-4) could be incorporated into sarcomeres in the *C. elegans* body wall muscle (Hayashi et al., 2019). However, several studies have indicated that GFP-actin does not behave in the same manner as untagged actin (Doyle and Botstein, 1996; Aizawa et al., 1997; Chen et al., 2012). Therefore, it would be desirable to assess other less invasive methods for *in vivo* actin labeling than direct actin tagging with a fluorescent protein. By comparing previously published strains, I found that while stable low expression of Lifeact causes no detectable effects on the sarcomeres, overexpression of Lifeact in the body wall muscle causes severe sarcomere disorganization and embryonic or larval lethality. Accordingly, Lifeact, at low concentrations, is a useful F-actin probe in *C. elegans* muscle, but artificial detrimental effects of Lifeact need to be carefully evaluated.

## Results

### Overexpression of Lifeact in the body wall muscle causes embryonic or larval lethality

**FIGURE 1:**
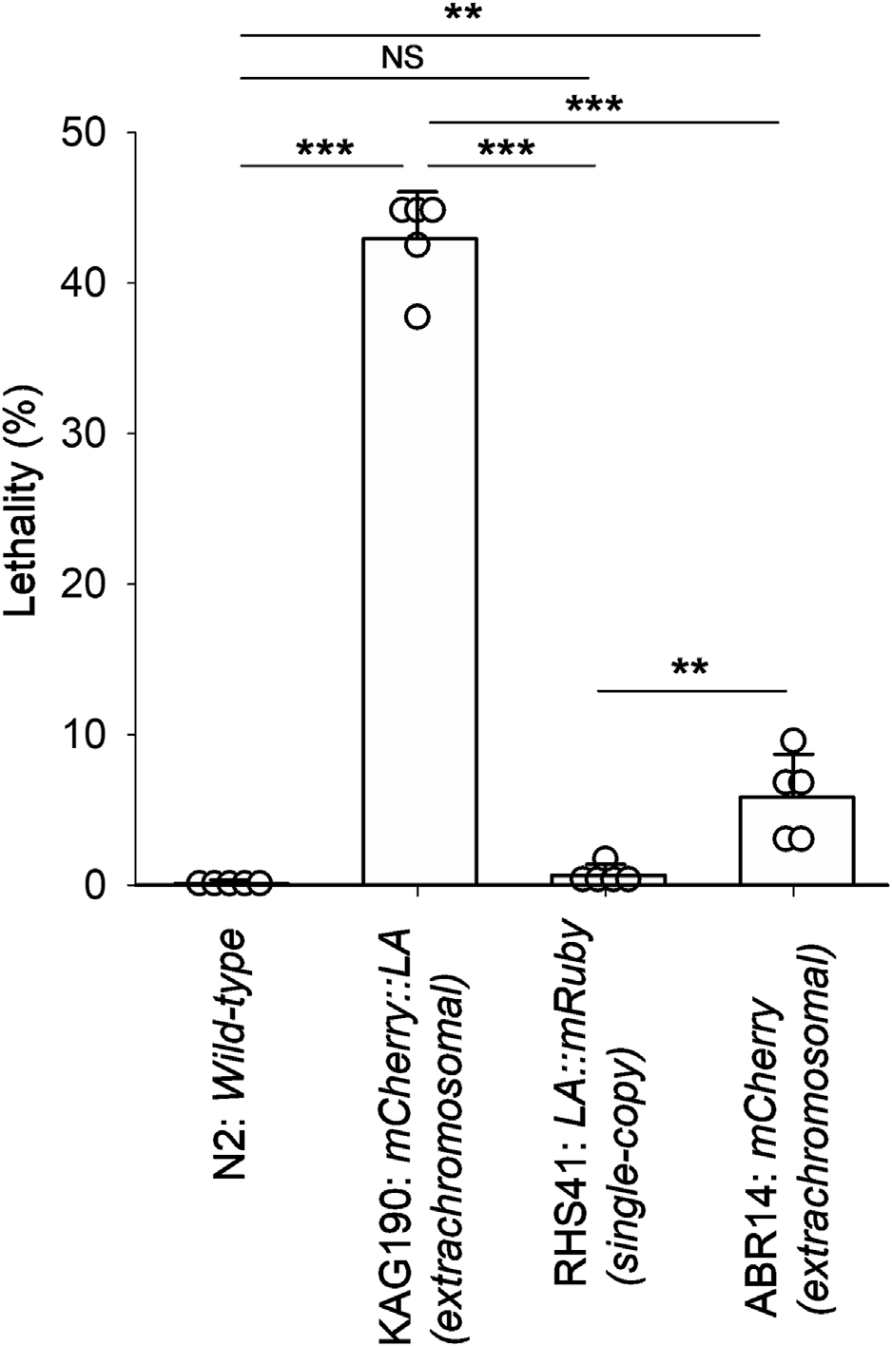
Expression of mCherry::Lifeact in the *C. elegans* body wall muscle from an extrachromosomal array causes high lethality. Lethality of embryos and larvae was quantified for N2 (wild-type), KAG190 [mCherry::Lifeact (abbreviated to LA) expressed from an extrachromosomal array], RHS41 (Lifeact::mRuby expressed from a single-copy transgene), and ABR14 (mCherry alone expressed from an extrachromosomal array). For transgenic strains, only animals with the expression of the fluorescent proteins were counted. Data are presented as average ± standard deviation (n = 5). **, 0.01 < P < 0.001. ***, P < 0.001. NS, not significant.

During the previous studies to localize CLIK-1, a calponin-related protein, in the sarcomeres of the *C. elegans* body wall muscle (Ono and Ono, 2020; Ono, 2024), we utilized a strain, KAG190, containing a transgene to express mCherry::Lifeact in the body wall muscle driven by the *myo-3* promoter (Brouilly et al., 2015). The *myo-3* gene encodes a myosin heavy chain that is predominantly expressed in the body wall muscle (Miller et al., 1983; Waterston, 1989; Okkema et al., 1993). This transgene was maintained as an extrachromosomal array that can contain a high copy number of the transgene and is often inherited in a mosaic manner within a transgenic animal (Mello et al., 1991; Mello and Fire, 1995). These characteristics can cause overexpression of a transgene and/or cell-to-cell variability in the expression levels. When mCherry::Lifeact was expressed at low levels in KAG190, it properly labeled sarcomeric actin filaments in the muscle cells (Brouilly et al., 2015; Ono, 2024). However, I also noticed a high occurrence of arrested embryos and L1 larvae when the expression of mCherry::Lifeact was high. Quantitation of lethality indicated that 43% ± 3.1% of mCherry::Lifeact-positive animals were arrested either as late embryos or L1 larvae (Figure 1). Most of the arrested embryos were elongated and immobile, suggesting that they failed to hatch. In contrast, wild-type (N2) animals with no transgene rarely produced arrested embryos or larvae (Figure 1). A strain that carries a single-copy transgene encoding Lifeact::mRuby with the *myo-3* promoter (RHS41) expressed uniformly low levels of Lifeact::mRuby throughout all body wall muscle cells (Higuchi-Sanabria et al., 2018) and rarely produced arrested embryos or larvae (Figure 1). As a control to examine the effect of mCherry, I examined ABR14 carrying a transgene in an extrachromosomal array to express mCherry alone by the *myo-3* promoter (Han et al., 2017). The lethality of this strain was slightly higher than that of N2 wild-type or RHS41 (single-copy Lifeact::mRuby) but much lower than that of KAG190 (mCherry::Lifeact overexpression) (Figure 1), indicating that overexpression of Lifeact caused lethality. These results indicate that overexpression of Lifeact in the body wall muscle is sufficient to cause embryonic or larval lethality.

### Overexpression of Lifeact in the body wall muscle causes sarcomere disorganization

**FIGURE 2:**
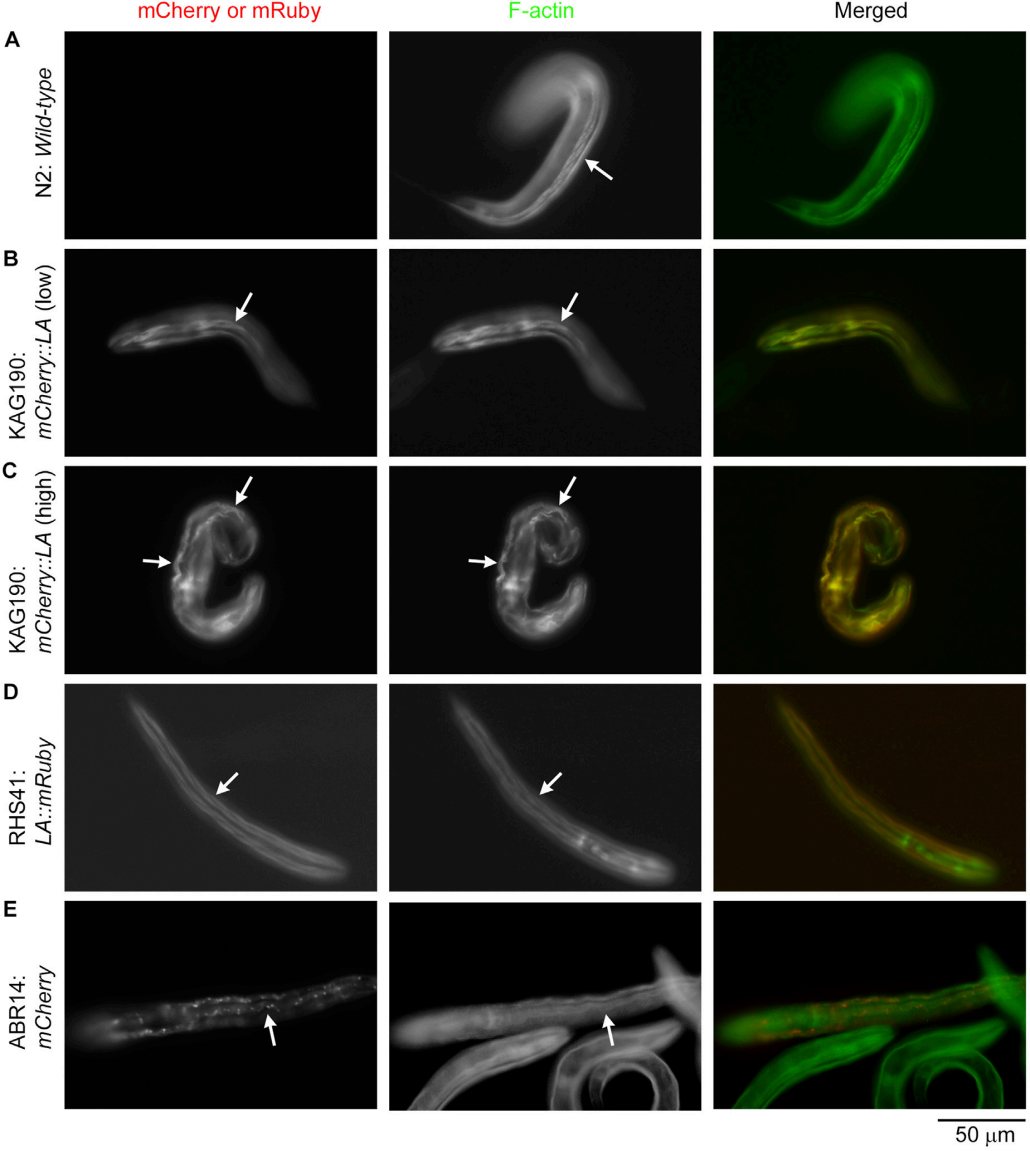
Overexpression of mCherry::Lifeact in the *C. elegans* body wall muscle causes severe disorganization of actin filaments in L1 larvae. L1 larvae of the indicated strains (Lifeact is abbreviated to LA) were stained with Alexa 488-phalloidin to visualize F-actin. Strains were N2 **(A)**, KAG190 [**(B)**: low expression; **(C)**: high expression], RHS41 **(D)**, and ABR14 **(E)**. Images shown are mCherry or mRuby (left), F-actin (middle), and merged (right: mCherry or mRuby in red; F-actin in green). Arrows indicate representative regions of the body wall muscle. Bar, 50 μm.

**FIGURE 3:**
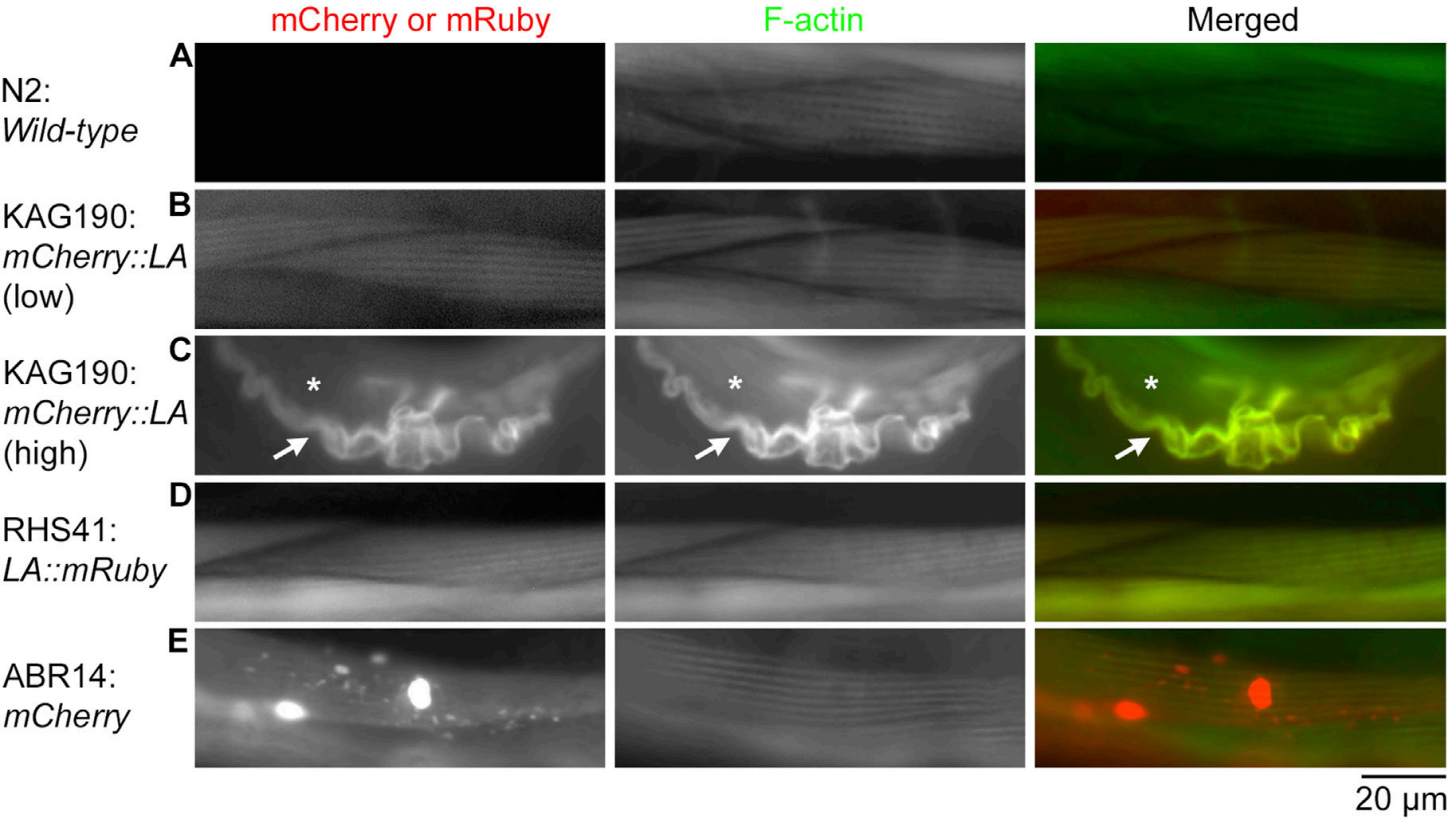
Overexpression of mCherry::Lifeact in the *C. elegans* body wall muscle causes severe disorganization of actin filaments in adult worms. Adult worms of the indicated strains (Lifeact is abbreviated to LA) were stained with Alexa 488-phalloidin to visualize F-actin, and their body wall muscle cells were examined. Strains were N2 **(A)**, KAG190 [**(B)**: low expression; **(C)**: high expression], RHS41 **(D)**, and ABR14 **(E)**. Images shown are mCherry or mRuby (left), F-actin (middle), and merged (right: mCherry or mRuby in red; F-actin in green). In panel C, arrows indicate a muscle cell that overexpressed mCherry::Lifeact, and asterisks indicate a neighboring muscle cell that did not express mCherry::Lifeact and had intact sarcomeric F-actin. Bar, 20 μm.

Examination of the actin filament organization showed that Lifeact overexpression caused severe sarcomere disorganization in the body wall muscle (Figures 2, 3). Although arrested embryos with mCherry::Lifeact overexpression showed highly disorganized actin filaments in the body wall muscle, these embryos were elongated and folded within confined eggshells, which made microscopic imaging of subcellular structures difficult. For clear comparison among the strains, I examined actin organization in the body wall muscle cells of L1 larvae (Figure 2) and adults (Figure 3) after staining with Alexa 488-labeled phalloidin. In wild-type L1 larvae, F-actin was normally accumulated into linear structures representing contractile apparatuses (Figure 2A, arrow). When the expression of mCherry::Lifeact was low, these F-actin structures were labeled, although some non-uniform distribution of F-actin was also detected (Figure 2B, arrows), suggesting minor disorganization. However, when the expression of mCherry::Lifeact was high, abnormal wavy F-actin bundles, which were heavily decorated by mCherry::Lifeact, were formed (Figure 2C, arrows). Lifeact::mRuby from a single-copy transgene was uniformly expressed in all body wall muscle cells at low levels and properly labeled F-actin structures without a detrimental effect (Figure 2D, arrows). When mCherry alone was overexpressed, aggregates of mCherry were formed, but sarcomeric actin organization was not visibly affected (Figure 2E, arrows).

Similarly, in adult worms, low-level expression of mCherry::Lifeact from the extrachromosomal array in KAG190 (Figure 3B) and Lifeact::mRuby from a single-copy transgene in RHS41 (Figure 3D) labeled F-actin in a striated sarcomeric pattern without disturbing the structure (compare with wild-type in Figure 3A). However, when mCherry::Lifeact was expressed at high levels, abnormally thick F-actin bundles containing mCherry::Lifeact were formed with no detectable sarcomere formation (Figure 3C). Because *C. elegans* body wall muscle cells are mononucleated and do not fuse, F-actin bundles are present only in the cell overexpressing mCherry::Lifeact (Figure 3C, arrow) but not in the neighboring cell without the expression of mCherry::Lifeact (Figure 3C, asterisk). When mCherry alone was overexpressed, aggregates of mCherry were formed with no detectable alterations in sarcomeric actin organization (Figure 3E). Therefore, overexpression of Lifeact in striated muscle cells is highly detrimental to the sarcomeric actin assembly, suggesting strongly that the muscle defects are the major cause of lethality.

## Discussion

Overexpression of mCherry::Lifeact in *C. elegans* body wall muscle caused severe disorganization of sarcomeric actin filaments and embryonic/larval lethality. This is consistent with the previous reports that severe defects in the sarcomeres of body wall muscle cause lethality at a late embryonic or L1 larval stage with defective body elongation (Waterston, 1989; Barstead and Waterston, 1991; Williams and Waterston, 1994; Ono et al., 2020). Since contraction of the body wall muscle is required for the body-axis elongation of *C. elegans* embryos (Zhang et al., 2011; Lardennois et al., 2019), severe sarcomere disorganization by Lifeact overexpression may prevent muscle contraction and affect embryonic and larval development. Although effects of overexpressed Lifeact on sarcomere components other than F-actin were not analyzed in this study, our previous studies indicate that disorganization of sarcomeric actin filaments causes relatively weak structural alterations in dense bodies and myosin thick filaments (Ono et al., 2003; Ono et al., 2020). Within the muscle cells, an antagonistic effect of Lifeact on cofilin in actin filament severing (Courtemanche et al., 2016) is expected to be impactful to sarcomere disorganization because UNC-60B, a muscle-specific actin depolymerizing factor/cofilin isoform, is essential for the assembly of sarcomeric actin filaments in the *C. elegans* body wall muscle (Ono et al., 1999; Ono et al., 2003). A recent structural study also showed that the Lifeact-binding site on F-actin overlaps with binding sites for cofilin and myosin (Belyy et al., 2020), suggesting that actomyosin contractility may be affected by Lifeact.

Aggregation of overexpressed mCherry may contribute to enhancing the artificial effects of Lifeact. Aggregate formation of mCherry has also been reported in cultured mammalian neurons (Ning et al., 2022). When multiple mCherry::Lifeact molecules associate, actin filaments can be bundled together and then recruit additional mCherry::Lifeact. Whether mRuby is also prone to aggregation remains unknown. In addition to the stabilizing effects of Lifeact on F-actin (Courtemanche et al., 2016), multimerization or aggregation of the tagged fluorescent protein is a factor that needs to be considered to avoid unfavorable effects on the actin cytoskeleton. Nonetheless, despite the fact that overexpressed Lifeact causes disorganization of the actin cytoskeleton, properly managed Lifeact is still considered one of the most useful probes for actin filaments, given that artificial effects of Lifeact and/or a fluorescent protein are carefully assessed.

## Materials and methods

### *C. elegans* strains and culture

The worms were cultured at 20°C, following standard methods using *Escherichia coli* OP50 for feeding (Stiernagle, 2006). Wild-type N2, ABR14 *shEx34 [myo-3p::mCherry]* (Han et al., 2017), and RHS41 *uthSi7 [myo-3p::Lifeact::mRuby]* (Higuchi-Sanabria et al., 2018) were obtained from the *Caenorhabditis* Genetics Center (Minneapolis, MN). KAG190 *Ex[myo-3p::mCherry::Lifeact; myo-2p::mCherry]* (Brouilly et al., 2015) was obtained from Kathrin Gieseler (University of Lyon, Villeurbanne, France) and maintained by picking mCherry-positive worms.

### Quantification of embryonic and larval lethality

Three to five gravid adult hermaphrodites were placed in an *E. coli* OP50-seeded 60-mm Nematode Growth Medium plate for 24 h and were let to lay eggs. After removing the adult worms, the remaining embryos and larvae on the plate were cultured for an additional 24 h and scored for dead or arrested embryos and larvae and healthy larvae (83–219 animals per plate, n = 5). For transgenic strains, only animals with the expression of the fluorescent proteins were counted. Since L1 larvae normally hatch ∼9 h after eggs are laid on the plate (Sulston et al., 1983), healthy worms are normally developed into the L2, L3, or L4 stage at the time of quantification. Data were analyzed by one-way analysis of variance with the Holm–Sidak method for pairwise comparison using SigmaPlot 15.0 (Grafiti LLC).

### Fluorescence microscopy

Staining of whole worms with Alexa 488-phalloidin (Thermo Fisher Scientific, catalog # A12379) was performed, as described previously (Ono, 2001; Ono, 2022). Samples were observed by epifluorescence using a Nikon Eclipse TE2000 inverted microscope (Nikon Instruments, Tokyo, Japan) with a CFI Plan Fluor ELWD 40× (dry; NA 0.60) objective. Images were captured using a Hamamatsu ORCA Flash 4.0 LT sCMOS camera (Hamamatsu Photonics, Shizuoka, Japan) and processed by NIS-Elements (Nikon Instruments) and Adobe Photoshop CS3 (Adobe).

## Data Availability

The original contributions presented in the study are included in the article/supplementary material; further inquiries can be directed to the corresponding author/s.
